# Laparoscopic Approach of a Unicornuate Uterus with Noncommunicating Rudimentary Horns

**DOI:** 10.5402/2011/906138

**Published:** 2010-10-07

**Authors:** Lidia Rosi Medeiros, Daniela Dornelles Rosa, Fabio Rosa Silva, Bruno Rosa Silva, Maria Ines Rosa

**Affiliations:** ^1^Departament of Gynecologic Surgery, Hospital Mãe de Deus, José de Alencar 1244 apt 1009, Porto Alegre 90880-480, RS, Brazil; ^2^Postgraduate Program in Medicine: Medical Sciences at Federal University of Rio Grande do Sul, Porto Alegre 90040-060, RS, Brazil; ^3^Laboratory of Epidemiology and National Institute for Translational Medicine, Postgraduate Program in Health Sciences, Health Sciences Unit, University of Southern Santa Catarina, 88806-000 Criciúma, SC, Brazil

## Abstract

*Background*. Müllerian duct malformations delineate a miscellaneous group of congenital anomalies that result from arrested development, abnormal formation, or incomplete fusion of the mesonephric ducts. *Case*. This paper describes the diagnosis and management of a noncommunicating rudimentary horn complicated by severe pelvic pain and associated endometriosis. *Conclusion*. This condition was diagnosed by laparoscopy and hysteroscopy examination. Operative videolaparoscopy proved to be a successful approach for the treatment of this congenital Müllerian anomaly.

## 1. Introduction

Müllerian duct malformations delineate a miscellaneous group of congenital anomalies that result from arrested development, abnormal formation, or incomplete fusion of the mesonephric ducts [1]. The incidence of uterine anomalies in a fertile population is reported to be around 3.2% [2]. The unicornuate uterus results from normal differentiation of the Müllerian duct, but a rudimentary functional horn may be found [3]. Patients with an asymmetric uterus and a rudimentary horn constitute 5% to 10% of those with uterovaginal anomalies [4]. Approximately 75% of such horns do not communicate with the normal hemiuterus [5]. Vaginal obstruction is associated with perivaginal mass, pain, and endometriosis, but cyclic menstrual flow may be present because of the normally functioning opposite side [4]. This anomaly is usually associated with ipsilateral renal agenesis (67%) or ipsilateral pelvic kidney [6].

## 2. Case

**Figure 1:**
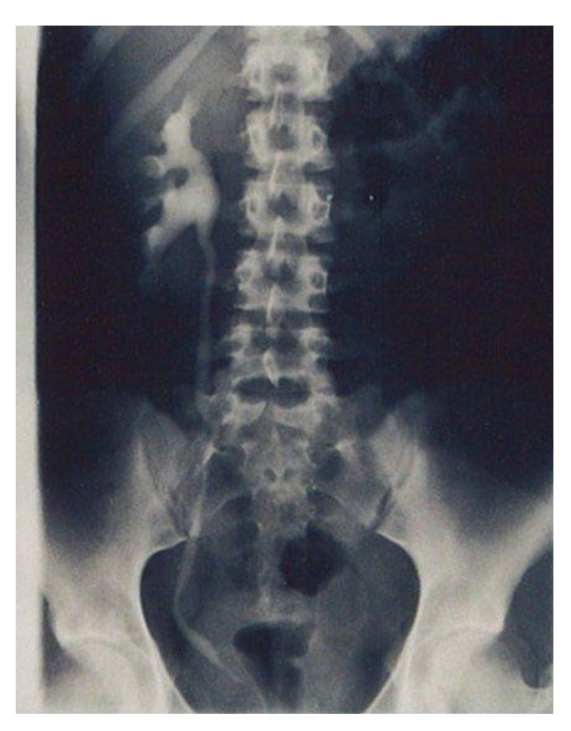
Urogram revealed absence of the left kidney.

**Figure 2:**
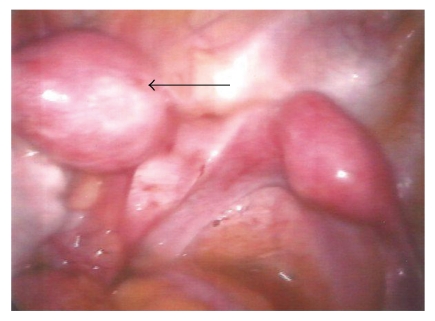
Normal right hemiuterus, tube, and ovary, and a left rudimentary uterine horn (arrow).

A 16-year-old nulliparous woman presented with severe dysmenorrhoea since menarche in November 2006 which was only minimally relieved with oral contraceptives and nonsteroidal anti-inflammatory drugs (NSAIDs). In April 2007, the patient experienced an episode of severe pain in the left lower quadrant of the abdomen. Pelvic ultrasound revealed a large irregular complex mass in the left hemipelvis with multiple cystic and solid components. The uterus and right ovary were thought to be normal. The left ovary could not be identified. The patient underwent diagnostic laparoscopy with hysteroscopy. Hysteroscopy showed a right unicornuate uterus and revealed a patent right cornus with no sign of ostium on the left side. Laparoscopy showed a right unicornuate uterus with a normal adnexa, a left non-communicating rudimentary horn (4 × 3 × 2 cm) with an enlarged and thickened tube, and a left ovarian endometrioma of 6 cm. A large endometrial cyst was washed out with irrigation fluid, and a biopsy was taken. After washing, the interior wall of the cyst was carefully examined to confirm the absence of intracystics lesions suspected to be malignant. The interior wall of the cyst was then destroyed using bipolar coagulation. Additionally, fibrous adhesions involving the ascending colon and small intestine were destroyed. Lysis of omental adhesions allowed identification of multiple areas of endometriosis in the posterior cul-de-sac, on the right and left uterosacral ligaments. There were no external genital abnormalities. A subsequent urogram revealed absence of the left kidney (Figure 1). Medical treatment for endometriosis using 6 months of gonadotropin-releasing hormone (GnRHa) was done. After extensive discussion with the patient laparoscopic removal of the left horn was indicated. The second laparoscopic examination was performed on September 2007. A four-puncture laparoscopy was performed with a 10-mm infraumbilical port, a 10 mm suprapubic port, and two 5-mm suprapubic ports laterally in the right abdominal side and in midline. Laparoscopy revealed a normal right hemiuterus, tube, and ovary, and a left rudimentary uterine horn (Figure 2). A left salpingectomy was started at the fimbriated end using bipolar coagulation and laparoscopic scissors. The left tube was used to pull up the rudimentary horn. The left uterus was dissected apart from the bladder using scissors and bipolar coagulation and was removed using a morcellator (Karl Storz, Germany). She went home 1 day after surgery and began a regimen of oral contraception with 75 *μ*g of desogestrel. She continues free of pelvic pain one year after surgery.

## 3. Comment

Around 75%–90% of cases of unicornuate uterus with rudimentary horn are non-communicanting [7]. The management of the present case illustrates the value of simultaneous laparoscopic and hysteroscopy evaluation of known uterine abnormalities. The literature suggests the need to remove the rudimentary horn of a unicornuate uterus and supports the laparoscopic approach if such a decision is taken [8–11]. A high incidence of associated endometriosis has been documented in cases of obstructive müllerian anomalies [8–11]. In the present case, the procedure was effective in resolving the pelvic pain and dysmenorrhea and avoided the risk of endometriosis. A GnRH agonist was used preoperatively in this case to reduce the vascularization and the inflammation that are often present around endometriotic lesions, facilitating surgical procedures [12]. 

In conclusion, operative laparoscopy is an excellent alternative to laparotomy for the management of unicornuate uterus with non-communicanting rudimentary horn. Commonly accepted benefits of minimally invasive surgery are enhanced visualization of the cul-de-sac, less adhesion formations, smaller incisions, reduced postoperative pain, and shortened hospital stay.
